# Supplementary material for: The interactome of intact mitochondria by cross-linking mass spectrometry provides evidence for coexisting respiratory supercomplexes
Source: Mol Cell Proteomics. 2017 Dec 8;17(2):216–32. doi: 10.1074/mcp.RA117.000470 (PMC5795388; doi:10.1074/mcp.RA117.000470)
Supplement: Supplemental Data [file supp_RA117.000470_133922_0_supp_23978_fzffwf.zip › spectra_annotation/mito_spectra_annotation/80-1-2-1-8-1.pdf]

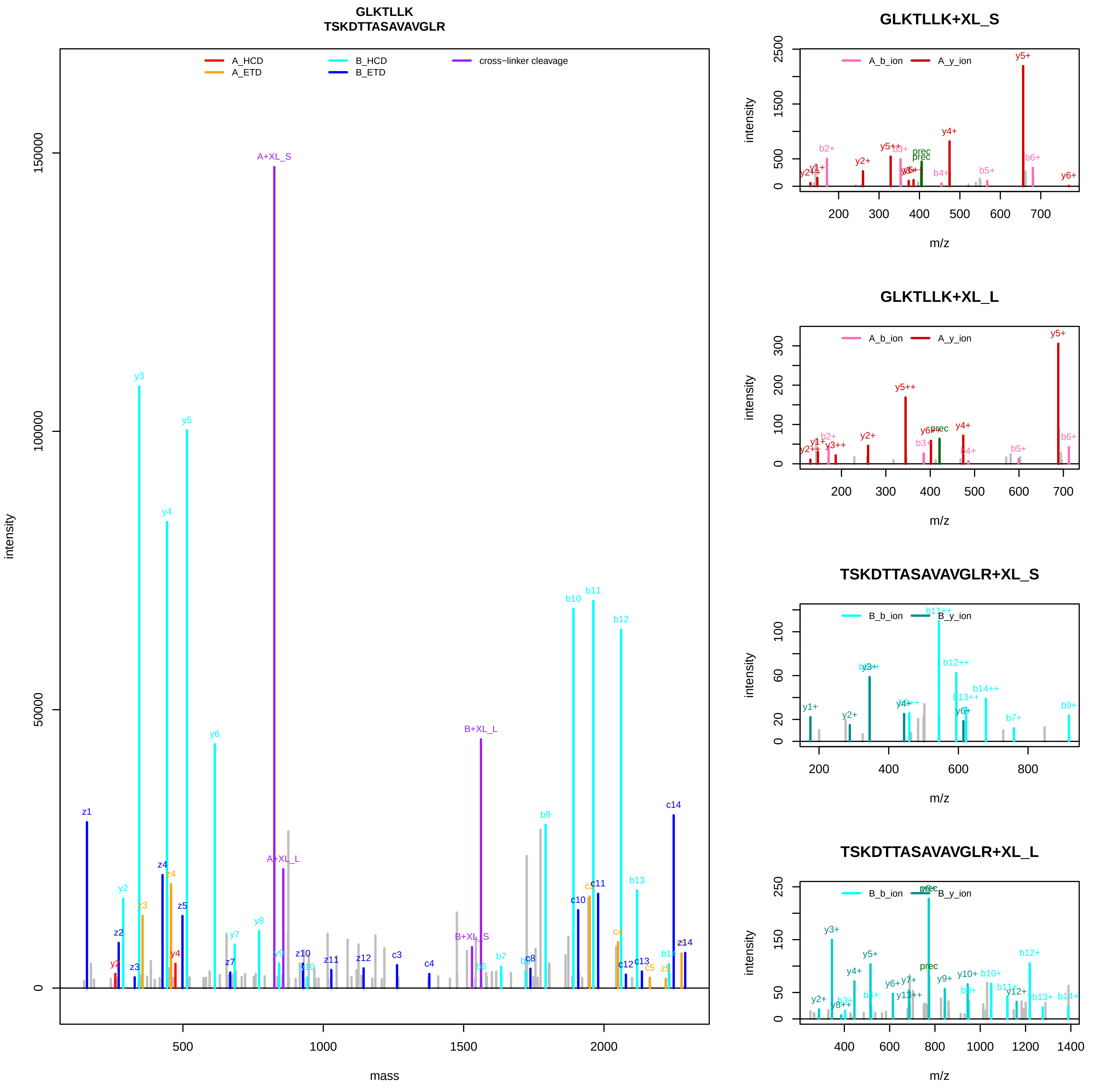
GLKTLLK
TSKDTTASAVAVGLR
A_HCD
A_ETD
B_HCD
B_ETD
cross−linker cleavage
A+XL_S
A+XL_L
B+XL_L
B+XL_S
y3
y4
y5
y6
y7
y8
y9
y10
y2
z1
z2
z3
z4
z5
z7
z10
z11
z12
z14
c3
c4
c5
c8
c10
c11
c12
c13
c14
b7
b9
b10
b11
b12
b13
intensity
mass
0
50000
100000
150000
500
1000
1500
2000
GLKTLLK+XL_S
A_b_ion
A_y_ion
y5+
y4+
y2+
b2+
b3+
b4+
b5+
b6+
y6+
y5++
prec
intensity
m/z
GLKTLLK+XL_L
y5++
y6++
y3++
TSKDTTASAVAVGLR+XL_S
B_b_ion
B_y_ion
b11++
b12++
b13++
b14++
y1+
y2+
y3+
y4+
y6+
b7+
b9+
TSKDTTASAVAVGLR+XL_L
y5+
y6+
y7+
y8++
y9+
y10+
y12+
y13++
b3+
b8+
b10+
b11+
b12+
b13+
b14+
